# Retrospective validation of bone risk stratification criteria for men with de novo metastatic hormone-naive prostate cancer in China

**DOI:** 10.7717/peerj.14500

**Published:** 2023-01-04

**Authors:** Yang Zhang, Li Ding, Yuxin Zheng, Kun Wang, Wentao Xia, Junqi Wang, Peng Ge

**Affiliations:** Department of Urology, the Affiliated Hospital of Xuzhou Medical University, Xuzhou, China

**Keywords:** Bone risk stratification, mHSPC, CRPC, Metastatic Hormone-Sensitive Prostate Cancer, China, PCa

## Abstract

**Background:**

Bone metastasis has been suggested to be a significant impactor on the prognosis of newly diagnosed de novo metastatic hormone-sensitive prostate cancer (mHSPC), and some risk stratification models have been proposed on the basis of this hypothesis. However, the effectiveness of these risk stratification criteria has not been fully evaluated in China. This study aimed to evaluate the effectiveness of the risk stratification models in China.

**Methods:**

A total of 140 patients who were newly diagnosed with metastatic prostate cancer followed by primary androgen deprivation-based therapy from January 2008 to June 2021 at our institution were enrolled in this study. The patients were divided into different groups on the basis of high- and low-volume disease (H/LVD) criteria, high-and low-risk disease (H/LRD) criteria, extremity bone metastasis criteria (EBM), and extent of disease (EOD) criteria. The area under the receiver operating characteristic (ROC) curve (AUC) and decision curve analysis (DCA) were used to compare the validity and net benefit of these models. Using the Cox proportional hazards model, we performed univariable and multivariable analyses of the factors influencing overall survival (OS) and the time of progression to metastatic castration-resistant prostate cancer (CRPC).

**Results:**

The median patient age was 72 years. Most patients had a Gleason score ≥8 (102 cases, 72.9%) and clinical T stage >2 (75 cases, 53.6%). The median follow-up time was 25 months (range, 2–95 months). Ninety-two patients progressed to CRPC and fifty-seven patients died during the follow-up. The AUC of OS and CRPC showed that the EOD model had higher validity than the other risk stratification models. DCA shows that the net benefit of the EOD model on OS was better than that of the other risk stratification models. As for CRPC, the net benefit of the EOD model was second only to that of the H/LRD model when the threshold was <0.5; however, when the threshold was >0.5, the EOD model outperformed the other models. The effectiveness of EOD as an independent prognostic variable was verified through univariable and multivariable analyses.

**Conclusion:**

The EOD model yields reasonable risk stratification for use in Chinese mHSPC patients, providing further evidence supporting its role in clinical decision-making.

## Introduction

Improvements in public awareness of prostate cancer (PCa) and the popularisation of prostate-specific antigen (PSA) examinations have resulted in early detection of increasing numbers of PCa cases. However, many cases still show de novo metastatic PCa (mPCa) at the time of diagnosis. According to the American Cancer Association, approximately 34,500 patients will die due to PCa (mainly metastatic stage) in the United States in 2022 (Siegel et al., 2022). mPCa is incurable and the main treatment modality is androgen deprivation therapy (ADT) or ADT-based therepy (Klotz, 2008). However, the prognosis of patients treated with ADT varies considerably, indicating significant individual differences in the status of metastatic hormone-sensitive prostate cancer (mHSPC).

The most common metastatic site of PCa is the bone. The prognosis of bone metastases in different sites and the number of bone metastases varies greatly (Suzuki et al., 2021). Soloway et al. (1988) proposed an extent of disease (EOD) grading system for patients with mHSPC treated with ADT, which was based on the number of bone metastases detected on bone scans; in this system, the patients were classified into five groups according to tumor grade: Grade 0 = normal imaging findings or imaging appearance of benign bone disease; Grade 1 < 6 bone metastases, with each metastasis involving less than 50% of the vertebral body (bone lesions more than 50% of the vertebral body were counted as two lesions); Grade 2 = 6–20 bone metastases; Grade 3 = lesion size >20 but less than a “super scan”; and Grade 4 = “super scan” or involvement of more than 75% of the ribs and vertebrae. Some researchers proposed the extremity bone metastases model (EBM) based on the sites of metastasis (Crawford et al., 1989; Eisenberger et al., 1998). Gravis et al. (2016) proposed high- and low-volume disease (H/LVD) models in the CHAARTED trial, taking into account the site and number of bone metastases. HVD was defined by the presence of visceral metastases and/or four bone lesions with at least one lesion outside the vertebral column and/or pelvis; otherwise, patients were defined as having LVD. Fizazi et al. (2017) proposed the concepts of high-and low-risk disease (H/LRD) in the LATITUDE trial based on the site and number of metastases and the Gleason score. Patients with at least two of the following three risk factors associated with poor prognosis were defined as having HRD: a Gleason score of 8 or higher, at least three bone metastases, and visceral metastases. Other patients were defined as having LRD. However, the significance of these risk stratification models in patients with mHSPC receiving ADT-based therepy has not been fully assessed due to the differences in the study populations enrolled in trials.

ADT-based therapy is more effective in Asian patients with mHSPC (Fukagai et al., 2006; Suzuki et al., 2020; Bernard et al., 2017; Cooperberg et al., 2016). The number of patients diagnosed with PCa in China is increasing annually (Liu et al., 2019; Akamatsu et al., 2019), with many patients showing bone metastases at the time of diagnosis. Therefore, we aimed to validate and compare the effectiveness of the four bone-related risk stratification criteria for patients with mHSPC treated with ADT-based therapy, in order to provide clinicians with more accurate prognostic tools to help clinical decision-making.

## Methods

### Patient information

This was a retrospective, single-institution study approved by the ethical committee of the Affiliated Hospital of Xuzhou Medical University (XYFY2022-KL192-01). A total of 140 patients who were newly diagnosed with metastatic prostate cancer from January 2008 to June 2021 at our institution were enrolled in this study. All patients were pathologically confirmed with prostate adenocarcinoma. All patients had complete imaging data and showed bone metastasis at the time of diagnosis. Nine patients also had visceral metastasis. ADT-based therapies were the initial hormonal therapy for these patients.In this study, ADT refers to surgical or medical castration. Among those, 113 received ADT+bicalutamide; seven received ADT+bicalutamide+docetaxel; 13 received ADT+abiraterone; and seven received ADT+flutamide as the primary treatment. In addition, these patients subsequently did not receive curative surgical treatment.

### Data collection

The clinicopathological data were retrospectively extracted from the medical charts, such as age, PSA level, alkaline phosphatase (ALP) level, haemoglobin (Hb) level, fibrinogen (Fib) level, clinical T stage (cT), Gleason score, and so on. The tumour-node-metastasis (TNM) stage was assigned according to the American Joint Committee on Cancer (Buyyounouski et al., 2017). The ^99m^Technetium-methylene-diphosphate (^99m^Tc-MDP) bone scintigraphy scan was performed before therapy. All the images were re-reviewed by the same person (author Yang Zhang) and the sites and number of bone metastases was recorded.

### Follow-up

The patients were followed up mainly by telephone, in combination with outpatient and/or inpatient visits. All data were entered into a password-protected database developed in Microsoft Access (version 2019). The endpoints were overall survival (OS) and the time of progression to metastatic castration-resistant prostate cancer (TTCRPC). The duration was calculated from the date of puncture biopsy of the prostate. The definitions of CRPC and OS were based on the recommendation (Cornford et al., 2017). The last follow-up date was July 4, 2022.

### Statistical analyses

Numerical variables was converted into binary variables according to reference range of the normal upper or lower limit and/or previous article classification standards (Fizazi et al., 2017; Narita et al., 2019; Tian et al., 2020). The area under the receiver operating characteristic (ROC) curve (AUC) analysis and decision curve analysis (DCA) were performed to compare the four risk stratification models and show the best discrimination and net benefit. In addition, univariable and multivariable Cox proportional hazards regression models were used to estimate the hazard ratio (HR) and corresponding 95% confidence interval (CI). A two-sided *P* < 0.050 was considered to be statistically significant. Analyses were performed using Statistical Product and Service Solutions software (SPSS, version 25.0), and R software (v.4.1.3, R Core Team, 2022).

## Results

### Patient characteristics and distribution of risk stratifications

A total of 140 patients with mHSPC were included in this study. Table 1 shows the clinicopathological characteristics of the study population. The median patient age was 72 years. Most patients showed Gleason score ≥ 8 (102 cases, 72.9%) and clinical T stage >2 (75 cases, 53.6%). The follow-up period was 2–95 months, with a median follow-up time of 25 months. Ninety-two patients progressed to CRPC and fifty-seven patients died during the follow-up.

**Table 1:** Patient characteristics.

Variables	All	H/LVD	H/LRD	EBM	EOD	
No. of patients	140	Low: 29	Low: 46	Yes: 92	EOD1: 36	EOD2: 42
		High: 111	High: 94	No: 48	EOD3: 29	EOD4: 33
Age (years) (range)	72(48–91)	Low: 77(57-87)	Low: 73(48-87)	Yes: 73(48-91)	EOD1: 77(57-87)	EOD2: 70(57-89)
		High: 71(48–91)	High: 72(52-91)	No: 72(57–87)	EOD3: 69(48–80)	EOD4: 73(52-91)
PSA (ng/mL) (range)	163.9(0.1–7125.0)	Low: 94.2(16.3-998.8)	Low: 157.9(16.3-3998.0)	Yes: 405.4(0.1-7125.0)	EOD1: 71.9(15.0-998.8)	EOD2: 154.8(0.1-3998.0)
		High: 299.7(0.1–7125.0)	High: 173.6(0.1–7125.0)	No: 91.1(16.3–3998.0)	EOD3: 404.6(30.2–2086.1)	EOD4: 601.0(16.0-7125.0)
Hb (g/dL) (range)	125(53-160)	Low: 133(81-158)	Low: 132(79-158)	Yes: 122(53-151)	EOD1: 137(81-159)	EOD2: 127(57-160)
		High: 124(53–160)	High: 124(53–160)	No: 134(81–160)	EOD3: 124(53–151)	EOD4: 119(71-148)
ALP (U/L) (range)	144(28–3301)	Low: 73(41-338)	Low: 100(41-3301)	Yes: 242(28-3301)	EOD1: 73(28-338)	EOD2: 98(63-989)
		High: 195(28–3301)	High:168(28–2732)	No: 84(41–338)	EOD3: 290(52–2698)	EOD4: 410(109-3301)
Fib(g/l)(range)	3.9(1.8–8.5)	Low: 3.4(2.2-6.6)	Low: 3.8(1.8-8.5)	Yes: 4.1(1.8-8.5)	EOD1: 2.2(3.4-6.6)	EOD2: 3.8(2.2-8.5)
		High: 4.1(1.8–8.5)	High: 4.1(2.2–7.6)	No: 3.7(2.2–6.6)	EOD3: 4.1(2.7–8.4)	EOD4: 4.5(1.8-7.6)
No. of patients with Visceral metastases	9	Low: 0	Low: 0	Yes: 4	EOD1: 3	EOD2: 3
		High: 9	High: 9	No: 5	EOD3: 3	EOD4: 0
No. of patients with Gleason score ≥ 8	102	Low: 83	Low: 9	Yes: 70	EOD1: 26	EOD2: 30
		High: 19	High: 93	No: 32	EOD3: 19	EOD4: 27
No. of patients with Clinical T stage >2	75	Low: 17	Low: 20	Yes: 47	EOD1: 22	EOD2: 23
		High: 58	High: 55	No: 28	EOD3: 14	EOD4: 16

**Notes.**

H/LVD high- and low-volume disease H/LRD high- and low-risk disease EBM extremity bone metastases model EOD extent of disease PSA prostate-specific antigen Hb hemoglobin ALP alkaline phosphatase Fib fibrinogen

According to the CHAARTED criteria, 111 patients had HVD, while 29 had LVD. According to the LATITUDE criteria, 94 had HRD, and 46 had LRD. According to the status of bone metastases in the extremities, there were 92 and 48 patients with and without bone metastases in the extremities, respectively. According to the EOD criteria, 36, 42, 29, and 33 patients had EOD grades 1, 2, 3, and 4, respectively.

### AUC analysis

**Figure 1:**
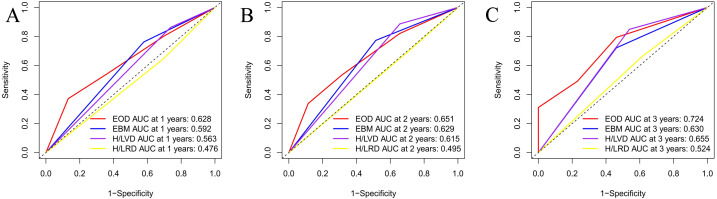
(A–C) Comparison of CRPC AUC analysis of each risk stratification.

**Figure 2:**
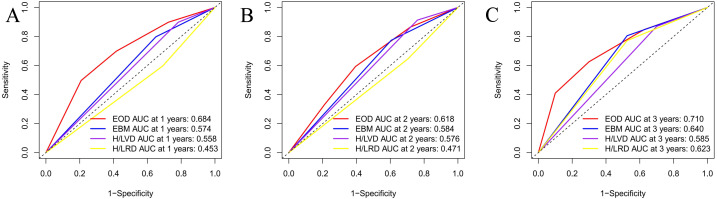
(A–C) Comparison of OS AUC analysis of each risk stratification.

An area under the curve (AUC) analysis of the four risk stratification systems was performed based on the time to CRPC and time to OS, and the findings of these analyses are illustrated in Figs. 1 and 2. Fig. 1 shows the CRPC-based comparison of the four risk stratification models (EOD, EBM, H/LVD, and H/LVD) on the basis of their CRPC AUCs. Compared to the three other models, EOD showed higher 1-, 2-, and 3-year CRPC AUC values. Fig. 2 provides a OS-based comparison. Similar to the results of CRPC, EOD also showed a higher 1-, 2-, and 3-year OS AUC value.

### DCA analysis

**Figure 3:**
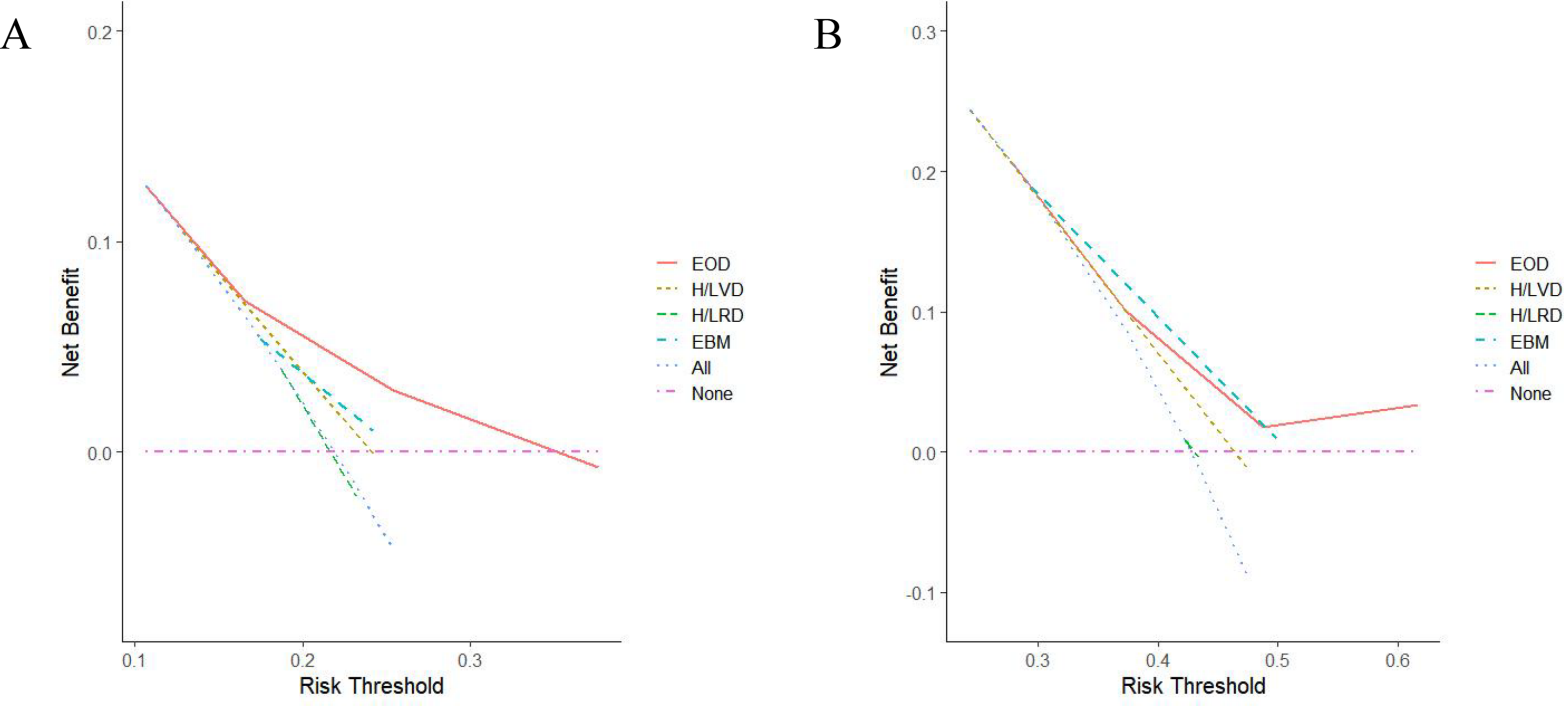
(A–C) Comparison of DCA analysis of each risk stratiûcation (A, OS; B, CRPC).

The DCA curves for the four models were plotted according to the time to CRPC and OS (Fig. 3). As shown in Fig. 3A, the net benefit of the EOD model on OS was better than those of the other risk stratification models. As for CRPC, the net benefit of the EOD model was second only to that of the H/LRD model when the threshold was <0.5; when the threshold was >0.5, the EOD model outperformed the other models (Fig. 3B).

### Analysis of the prognostic factors for OS and CRPC

In the univariable analysis, Hb level (<130 g/L v.s. ≥130 g/L; HR 1.846, 95% CI [1.060–4.075], *P* = 0.03), ALP level (>128 U/L v.s. ≤128 U/L; HR 2.340, 95% CI [1.344–4.075], *P* = 0.003), Fib level (>4 v.s. ≤4; HR 2.028, 95% CI [1.200–3.428], *P* = 0.008) and EOD ( ≥2 v.s. <2; HR 3.078, 95% CI [1.423–6.659], *P* = 0.004) were statistically significant on OS (Table 2). Since the ALP level showed collinearity with EOD, the multivariable analysis was not performed for this factor. In the multivariable analysis, EOD (≥2 vs. <2; HR 3.353, 95% CI [1.361–8.259], *P* = 0.009) was the independent factor influencing the OS. Similar results were found in univariable and multivariable analyses for CRPC (Table 3). H/LVD, H/LRD and EBM models were not good impactor for prognosis (Supplementary Material S1).

**Table 2:** Univariable and multivariable analyses of factors associated with the OS.

	Univariable				Multivariable			
			95%Cl				95%Cl	
Variables	*P* value	HR	Lower	Upper	*P* value	HR	Lower	Upper
Age >65 vs. ≤65(years)	0.203	1.591	0.779	3.253	0.044	2.202	1.021	4.751
PSA >100 vs. ≤100(ng/mL)	0.906	0.967	0.550	1.699	0.119	0.611	0.329	1.136
Hb <130 vs. ≥130(g/L)	0.030	1.846	1.060	4.075	0.180	1.491	0.831	2.674
ALP >128 vs. ≤128(U/L)	0.003	2.340	1.344	4.075	–	–	–	–
Fib >4 vs. ≤4(g/L)	0.008	2.028	1.200	3.428	0.062	1.701	0.973	2.974
Visceral metastases	0.897	1.080	0.336	3.472	0.219	2.188	0.629	7.614
Gleason score ≥8 vs. <8	0.848	1.057	0.598	1.871	0.512	0.819	0.451	1.487
Clinical T stage >2 vs. ≤2	0.334	1.295	0.766	2.188	0.037	1.831	1.038	3.230
EOD ≥2 vs. <2	0.004	3.078	1.423	6.659	0.009	3.353	1.361	8.259

**Notes.**

OS overall survival PSA prostate-specific antigen Hb hemoglobin ALP alkaline phosphatase Fib fibrinogen EOD extent of disease

**Table 3:** Univariable and multivariable analyses of factors associated with time to CRPC.

	Univariable				Multivariable			
			95%Cl				95%Cl	
Variables	*P* value	HR	Lower	Upper	*P* value	HR	Lower	Upper
Age >65 vs. ≤65(years)	0.979	0.993	0.610	1.618	0.928	1.024	0.611	1.717
PSA >100 vs. ≤100(ng/mL)	0.693	0.916	0.591	1.418	0.165	0.717	0.449	1.146
Hb <130 vs. ≥130(g/L)	0.864	0.965	0.639	1.456	0.406	0.830	0.535	1.288
ALP >128 vs. ≤128(U/L)	0.006	1.800	1.179	2.749	–	–	–	–
Fib >4 vs. ≤4(g/L)	0.039	1.545	1.023	2.334	0.175	1.344	0.877	2.061
Visceral metastases	0.345	0.616	0.226	1.682	0.571	0.744	0.267	2.071
Gleason score ≥8 vs. <8	0.419	0.829	0.526	1.307	0.189	0.727	0.452	1.170
Clinical T stage >2 vs. ≤2	0.445	1.174	0.777	1.774	0.149	1.378	0.891	2.129
EOD ≥2 vs. <2	0.016	1.896	1.127	3.189	0.007	2.254	1.245	4.080

**Notes.**

CRPC castration-resistant prostate cancer PSA prostate-specific antigen Hb hemoglobin ALP alkaline phosphatase Fib fibrinogen EOD extent of disease

### Subgroup analysis after excluding patients with visceral metastasis

In this study, all patients showed bone metastasis and nine patients also had visceral metastasis at the time of diagnosis. Since the number of patients with visceral metastasis is small, we conducted subgroup analyses on 131 patients only with bone metastasis to further verify our conclusions. The results were consistent (Supplementary Material S2).

## Discussion

In this study, we validated the four bone risk stratification criteria for men with de novo metastatic hormone-naive prostate cancer in China using AUC analysis and DCA. The results showed that the EOD model is more suitable than the H/LVD, H/LRD, and EBM models for Chinese patients with mHSPC treated with ADT-based therapy. The EOD model showed better performance in differentiation and a higher net benefit, and was an independent prognostic factor for CRPC and OS.

Racial differences are important consideration in the discussion of the reasons for the differences in prognosis between global and Chinese patients. Asian patients are more sensitive to hormone therapy in comparison with patients of other racial groups (Fukagai et al., 2006; Suzuki et al., 2020; Bernard et al., 2017; Cooperberg et al., 2016). Previous genomic analyses of PCa in China have revealed that the genomic alterations in Chinese patients are significantly different from those in Western patients (Li et al., 2020). ADT/ADT-based treatment is a “popular” treatment choice for metastatic prostate cancer. However, the prognosis of those patients varies considerably. In the LATITUDE trial, the addition of abiraterone acetate plus prednisone to ADT in patients with mHSPC resulted in a significant improvement in OS and progression-free survival in comparison with placebo and prednisone plus ADT (Fizazi et al., 2017). The CHAARTED trial showed that docetaxel systemic chemotherapy combined with ADT prolonged the OS of patients with HVD in comparison with ADT alone (Gravis et al., 2016). Totally, four bone risk stratification criteria are popularly used in clinical practice, i.e., H/LVD, H/LRD, EBM and EOD criteria. Studies are warranted to assess the clinical utility of the existing four risk stratification models for bone metastasis in patients with mHSPC. To the best of our knowledge, this was the first study to thoroughly validate and compare the prognostic significance of several bone risk stratification models based on different perspectives in Chinese patients.

Our study included 140 Chinese patients with mHSPC. The patients were followed up for relevant information on CRPC and OS. Comparative analysis of the AUC values of the H/LVD, H/LRD, EBM, and EOD models showed that the EOD model was significantly better in differentiation than the other models. Comparison of the DCA curves associated with OS for patients with mHSPC demonstrated that the EOD model had the highest net benefit. The DCA showed that H/LRD model had the highest net benefit when the threshold was <0.5, while the EOD model was second only to the H/LRD model. When the threshold was >0.5, the EOD model had the highest net benefit.

Bone metastatic burden is one of the important prognostic markers for patients with mHSPC (Akamatsu et al., 2019; Miyoshi et al., 2015). Several studies reported that the four models mentioned above were the independent risk factor affecting TTCRPC and OS (Narita et al., 2019; Kawahara et al., 2020). Similarly, our study comfirmed that EOD was an independent prognostic factor for TTCRPC and OS. However, of note, H/LVD, H/LRD and EBM were not the independent prognostic factors for OS in univariable and multivariable analyses. The HVD and patients with EBM group showed a shorter TTCRPC than the LVD and the patients without EBM group, respectively (Supplementary Material S1). One possible explanation is that these bone prognosis models may not be fully applicable to Chinese patients with mHSPC.

The biology of PCa is being studied worldwide and has potential clinical applications. However, the use of genetic markers as therapeutic targets is currently limited in clinical practice. Among clinical factors, the number and site of bone metastases, presence of visceral metastases, PSA level, testosterone level, and other treatments are considered important factors affecting the prognosis of patients with mHSPC (Tian et al., 2020; Hori et al., 2011). Similarly, in the univariable analysis, we found that Hb, ALP, Fib and EOD were statistically significant for OS (Table 2). In the multivariable analysis, EOD (≥2 v.s. <2; HR 3.353, 95% CI [1.361–8.259], *P* = 0.009) was still the independent factor influencing the OS. Collectively, EOD was a strong predictor for prognosis.

This study had several limitations. First, the retrospective nature of the study was an inherent limitation. Second, this study included data from a single centre and had a relatively small sample size. Therefore, multi-centre, large-sample data are required to confirm the findings of this study and to validate the accuracy of the model. Larger studies with longer follow-up periods are warranted to validate and compare the prognostic impact of different prognostic models. In addition, there is a need to further incorporate some of the newly developed drugs for ADT-based therepy, or to conduct a more detailed delineation of the suitability of the individual models for different drugs.

## Conclusion

The EOD model yields reasonable risk factors for use in Chinese mHSPC patients, providing further evidence supporting clinical decision-making.

##  Supplemental Information

10.7717/peerj.14500/supp-1 Supplementary Material S1 Supplementary Tables

10.7717/peerj.14500/supp-2 Supplementary Material S2 Supplementary Figures

10.7717/peerj.14500/supp-3 Data S1 Raw data

## Additional Information and Declarations

### Data Availability

The following information was supplied regarding data availability: The raw data is available in the Supplemental File.
