# Supplementary material for: Retrospective validation of bone risk stratification criteria for men with de novo metastatic hormone-naive prostate cancer in China
Source: PeerJ. 2023 Jan 4;11:e14500. doi: 10.7717/peerj.14500 (PMC9825052; doi:10.7717/peerj.14500)
Supplement: Supplementary Material S1 [file peerj-11-14500-s001.pdf]

Table S1: Univariable and multivariable analyses of factors associated with the OS

Overall survival	Univariable				Multivariable			
			95%Cl				95%Cl	
Variables	P value	HR	Lower	upper	P value	HR	Lower	upper
Age ＞65 vs. ≤65(years)	0.203	1.591	0.779	3.253	0.053	2.142	0.990	4.635
PSA > 100 vs. ≤100(ng/mL)	0.906	0.967	0.550	1.699	0.427	0.786	0.434	1.424
Hb ＜130 vs. ≥130(g/L)	0.030	1.846	1.060	4.075	0.057	1.753	0.984	3.126
ALP ＞128 vs. ≤128(U/L)	0.003	2.340	1.344	4.075	-	-	-	-
Fib > 4 vs. ≤4(g/L)	0.008	2.028	1.200	3.428	0.017	1.950	1.126	3.377
visceral metastases	0.897	1.080	0.336	3.472	0.330	1.843	0.539	6.302
Gleason score ≥8 vs. ＜8	0.848	1.057	0.598	1.871	0.494	0.813	0.448	1.473
Clinical T stage > 2 vs. ≤2	0.334	1.295	0.766	2.188	0.060	1.713	0.977	3.001
HRD vs. LRD	0.417	1.266	0.716	2.240	-	-	-	-
HVD vs. LVD	0.059	2.011	0.975	4.150	0.091	1.972	0.898	4.331
EBM Yes vs. No	0.204	1.439	0.820	2.526	-	-	-	-

OS: overall survival; PSA: prostate-specific antigen; Hb: hemoglobin ; ALP: alkaline phosphatase ; Fib: fibrinogen;EOD: extent of disease.

Table S2: Univariable and multivariable analyses of factors associated with time to CRPC

Time to CRPC	Univariable				Multivariable				Multivariable			
			95%Cl				95%Cl				95%Cl	
Variables	P value	HR	Lower	upper	P value	HR	Lower	upper	P value	HR	Lower	upper
Age ＞65 vs. ≤65(years)	0.979	0.993	0.610	1.618	0.830	1.058	0.631	1.774	0.695	1.113	0.652	1.899
PSA > 100 vs. ≤100(ng/mL)	0.693	0.916	0.591	1.418	0.296	0.785	0.499	1.235	0.225	0.750	0.471	1.194
Hb ＜130 vs. ≥130(g/L)	0.864	0.965	0.639	1.456	0.532	0.872	0.568	1.340	0.416	0.834	0.538	1.292
ALP ＞128 vs. ≤128(U/L)	0.006	1.800	1.179	2.749	-	-	-	-	-	-	-	-
Fib > 4 vs. ≤4(g/L)	0.039	1.545	1.023	2.334	0.170	1.345	0.881	2.054	0.270	1.275	0.828	1.962
visceral metastases	0.345	0.616	0.226	1.682	0.215	0.522	0.187	1.458	0.630	0.778	0.281	2.154
Gleason score ≥8 vs. ＜8	0.419	0.829	0.526	1.307	0.111	0.679	0.421	1.093	0.080	0.650	0.401	1.054
Clinical T stage > 2 vs. ≤2	0.445	1.174	0.777	1.774	0.148	1.377	0.892	2.126	0.165	1.359	0.882	2.094
HRD vs. LRD	0.869	0.964	0.620	1.498	-	-	-	-	-	-	-	-
HVD vs. LVD	0.007	2.268	1.255	4.101	0.002	2.721	1.437	5.152	-	-	-	-
EBM Yes vs. No	0.004	0.511	0.322	0.812	-	-	-	-	0.002	2.301	1.356	3.902

CRPC: castration-resistant prostate cancer; PSA: prostate-specific antigen; Hb: hemoglobin ; ALP: alkaline phosphatase ; Fib: fibrinogen;EOD: extent of disease.
