# Supplementary material for: Retrospective validation of bone risk stratification criteria for men with de novo metastatic hormone-naive prostate cancer in China
Source: PeerJ. 2023 Jan 4;11:e14500. doi: 10.7717/peerj.14500 (PMC9825052; doi:10.7717/peerj.14500)
Supplement: Supplementary Material S2 [file peerj-11-14500-s002.pdf]

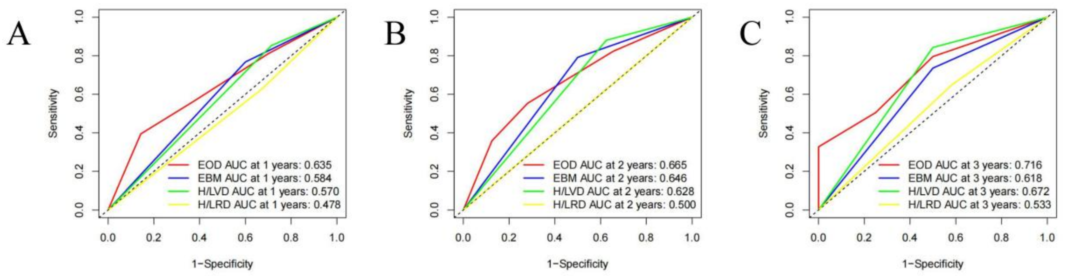

Figure S1. Comparison of CRPC AUC analysis of each risk stratification

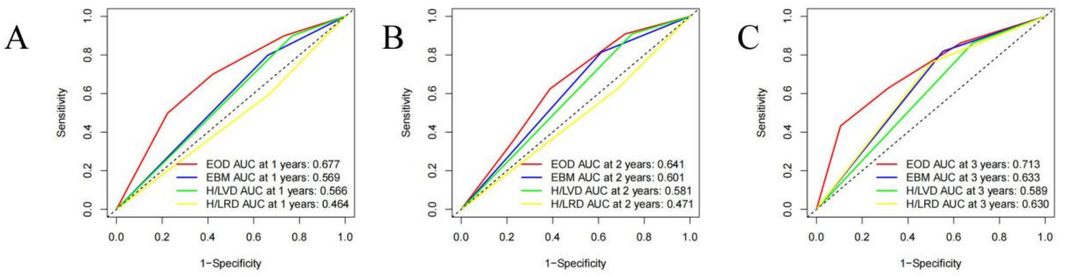

Figure S2. Comparison of OS AUC analysis of each risk stratification

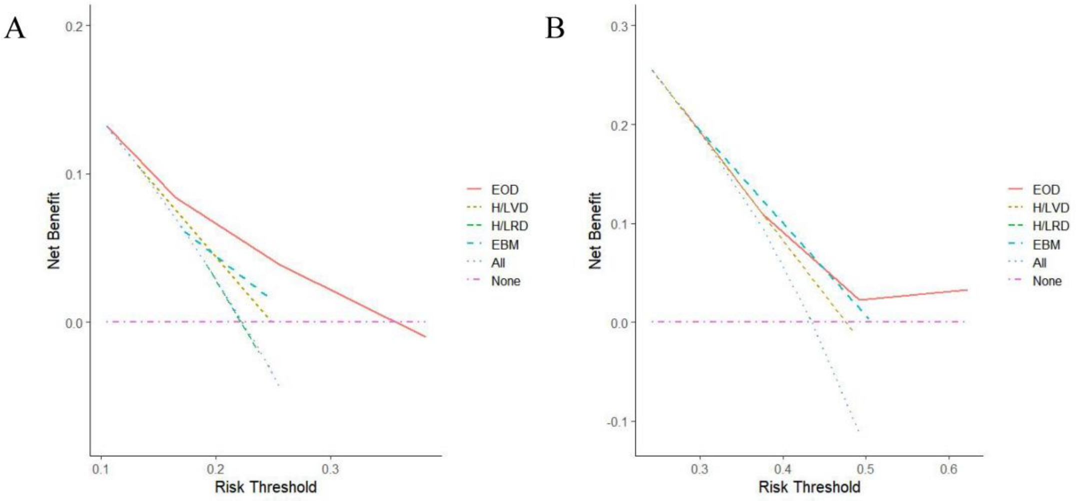

Figure S3. Comparison of DCA analysis of each risk stratiûcation (A:about OS,B:about CRPC) .

Table S3: Univariable and multivariable analyses of factors associated with the OS

Overall survival	Univariable				Multivariable			
			95%Cl				95%Cl	
Variables	P value	HR	Lower	upper	P value	HR	Lower	upper
Age >65 vs. ≤65(years)	0.254	1.551	0.730	3.296	0.059	2.177	0.972	4.876
PSA > 100 vs. ≤100(ng/mL)	0.929	1.027	0.570	1.852	0.203	0.656	0.343	1.256
Hb <130 vs. ≥130(g/L)	0.029	1.906	1.068	3.401	0.195	1.488	0.815	2.717
ALP >128 vs. ≤128(U/L)	0.002	2.433	1.376	4.300	-	-	-	-
Fib > 4 vs. ≤4(g/L)	0.010	2.027	1.181	3.479	0.094	1.634	0.920	2.903
Gleason score ≥8 vs. <8	0.674	1.134	0.630	2.042	0.700	0.887	0.481	1.635
Clinical T stage > 2 vs. ≤2	0.294	1.336	0.778	2.294	0.050	1.790	1.000	3.202
EOD ≥2 vs. <2	0.004	3.392	1.492	7.713	0.010	3.499	1.346	9.100

OS: overall survival; PSA: prostate-specific antigen; Hb: hemoglobin ; ALP: alkaline phosphatase ; Fib: fibrinogen;EOD: extent of disease.

Table S4: Univariable and multivariable analyses of factors associated with time to CRPC

Time to CRPC	Univariable				Multivariable			
			95%Cl				95%Cl	
Variables	P value	HR	Lower	upper	P value	HR	Lower	upper
Age >65 vs. ≤65(years)	0.976	1.008	0.606	1.677	0.830	1.060	0.620	1.814
PSA > 100 vs. ≤100(ng/mL)	0.955	0.987	0.627	1.555	0.315	0.779	0.479	1.267
Hb <130 vs. ≥130(g/L)	0.992	1.002	0.656	1.532	0.604	0.888	0.568	1.389
ALP >128 vs. ≤128(U/L)	0.006	1.835	1.192	2.825	-	-	-	-
Fib > 4 vs. ≤4(g/L)	0.093	1.435	0.941	2.188	0.260	1.288	0.830	1.999
Gleason score ≥8 vs. <8	0.394	0.819	0.517	1.296	0.162	0.710	0.439	1.148
Clinical T stage>2 vs. ≤2	0.637	1.107	0.726	1.687	0.258	1.292	0.829	2.014
EOD ≥2 vs. <2	0.022	1.870	1.096	3.192	0.017	2.096	1.143	3.843

CRPC: castration-resistant prostate cancer; PSA: prostate-specific antigen; Hb: hemoglobin ; ALP: alkaline phosphatase ; Fib: fibrinogen;EOD: extent of disease.
